# Current Overview of Metal Nanoparticles’ Synthesis, Characterization, and Biomedical Applications, with a Focus on Silver and Gold Nanoparticles

**DOI:** 10.3390/ph16101410

**Published:** 2023-10-04

**Authors:** Ana Flavia Burlec, Andreia Corciova, Monica Boev, Denisa Batir-Marin, Cornelia Mircea, Oana Cioanca, Gabriela Danila, Marius Danila, Anca Florentina Bucur, Monica Hancianu

**Affiliations:** 1 Faculty of Pharmacy, “Grigore T. Popa” University of Medicine and Pharmacy Iasi, 700115 Iasi, Romania; ana-flavia.l.burlec@umfiasi.ro (A.F.B.); maria.corciova@umfiasi.ro (A.C.); cornelia.mircea@umfiasi.ro (C.M.); oana.cioanca@umfiasi.ro (O.C.); monica.hancianu@umfiasi.ro (M.H.); 2 Research Centre in the Medical-Pharmaceutical Field, Faculty of Medicine and Pharmacy, “Dunarea de Jos” University of Galati, 800008 Galati, Romania; gd243@student.ugal.ro (G.D.); dumitru.danila@ugal.ro (M.D.); fb173@student.ugal.ro (A.F.B.)

**Keywords:** inorganic nanoparticles, biological synthesis, anticancer activity, antimicrobial activity, clinical trials

## Abstract

Metal nanoparticles (NPs) have garnered considerable attention, due to their unique physicochemical properties, that render them promising candidates for various applications in medicine and industry. This article offers a comprehensive overview of the most recent advancements in the manufacturing, characterization, and biomedical utilization of metal NPs, with a primary focus on silver and gold NPs. Their potential as effective anticancer, anti-inflammatory, and antimicrobial agents, drug delivery systems, and imaging agents in the diagnosis and treatment of a variety of disorders is reviewed. Moreover, their translation to therapeutic settings, and the issue of their inclusion in clinical trials, are assessed in light of over 30 clinical investigations that concentrate on administering either silver or gold NPs in conditions ranging from nosocomial infections to different types of cancers. This paper aims not only to examine the biocompatibility of nanomaterials but also to emphasize potential challenges that may limit their safe integration into healthcare practices. More than 100 nanomedicines are currently on the market, which justifies ongoing study into the use of nanomaterials in medicine. Overall, the present review aims to highlight the potential of silver and gold NPs as innovative and effective therapeutics in the field of biomedicine, citing some of their most relevant current applications.

## 1. Introduction

Over the past two decades, significant advancements in nanotechnology have been recorded, since researchers have discovered multiple ways to design and use nanostructured materials in a variety of fields (e.g., medicine, pharmacy, biology, the food and textiles industries, agriculture and electronics) [1].

The ability to control and direct the behavior of atoms and molecules is a key component of nanoscience. The etymology of the word “nano” has its origin in the Greek word “nanos”, which means “dwarf”; “nanotechnology” thus refers to particles with sizes between 1 and 100 nm, equivalent to three linearly aligned atoms’ lengths [2], dispersed in a solid, liquid or gaseous phase [3,4].

The unique characteristics of such particles, including their size, structure, and composition, make them useful as disinfectants, genetic vector and medication delivery systems, nano-magnets, or as water depollution vectors [5,6].

Nanoparticles (NPs) can vary largely in size. Generally, they can be *zero-dimensional*, meaning that their length, thickness and height are all determined by a single point, as in the case of nano dots; *one-dimensional*, meaning that they only present a single parameter, as in the case of nanotubes; *two-dimensional*, such as graphene; and *three-dimensional*, such as metal NPs (silver, gold, copper, etc.). NPs can also have a wide range of structures and shapes (e.g., spherical, cylindrical, tubular, conical, helical), while some can be flat or even irregular. NPs can be aerated or compact, amorphous or crystalline, with one or more solid crystals [7].

The uniqueness and specificity of such materials are usually granted by the synthesis method. Even a small change in the technological process can lead to major differences in their essential properties. After performing the synthesis through one of the two major routes, NPs should be characterized in terms of size, composition, electric surface charge and surface morphology. This characterization can be achieved using various techniques, that range from visual inspection to XRD (X-Ray Diffraction) analysis [8,9].

Following a general classification of nanomaterials, this review focuses on the synthesis, characterization, and biomedical applications, as well as on the cytotoxicity, of metal NPs, with a specific focus on silver and gold NPs. Such materials have the appealing capacity of improving the stability and solubility of encapsulated cargos, of facilitating transport across cell membranes, and of facilitating the delivery at specific levels [10].

Noble elements such as gold, silver, and palladium in the form of NPs represent some of the most promising trends in nanotechnology, especially for creating bioengineering materials that could be used as cutting-edge diagnostic tools and devices to treat severe diseases. For instance, silver and gold NPs generally hold considerable significance in the field of nanotechnology and materials science due to their unique properties and versatile applications. These noble metal NPs stand out for their exceptional optical, catalytic and antimicrobial properties, and effective targeted drug delivery in various types of cancers [11]. Their distinctive plasmonic properties enable them to manipulate light at the nanoscale, making them invaluable in areas such as photonics and sensor technology. Additionally, silver NPs are renowned for their potent antimicrobial properties, thus finding widespread use in medical applications and as disinfectants [12]. Gold NPs, on the other hand, exhibit remarkable catalytic activity and are employed in various chemical reactions and drug delivery systems [13]. Compared to other metal or metal oxide NPs, silver and gold NPs often excel in terms of stability and tunable surface chemistry. Furthermore, they are also well-known for their capacity to efficiently convert and manipulate energy and matter at nanoscale. These qualities make them indispensable tools for researchers seeking innovative solutions in fields ranging from medicine to environmental science [14,15]. Moreover, such NPs can be found in a variety of everyday items, such as personal care items, food-storage containers, cosmetics and bandages [8].

The significant interest in nanotechnology can also be seen in the number of results obtained when searching for keywords such as ‘nanoparticles’ or ‘metal nanoparticles’, with the latter search currently generating around 170,000 results in SpringerLink and 85,000 results in PubMed, respectively. References from major databases such as PubMed, Science Direct, SpringerNature, Scopus, SpringerLink, Google Scholar, and Web of Science were used to assemble this review. The novelty of the current paper lies in revealing the most recent advancements in the medical use of gold and silver NPs synthesized in various manners, including the most up-to-date incidents of such nanomaterials being included in in vitro, in vivo and clinical studies.

## 2. Classification of NPs

Nanomaterials can generally be classified depending on the employed synthesis technique and on the origin of the materials used in the process: organic, inorganic, or carbon-based [16]. The main categories of NPs with potential applications in biomedicine are briefly presented below, along with some promising nanomaterial combinations.

### 2.1. Organic Nanoparticles

Organic NPs are widely employed in the biomedical and pharmaceutical areas, as systems for the controlled release of medicinal substances, notably by injection at the level of specific organs [17]. This category mainly includes micelles, liposomes, dendrimers, polymeric and protein nanoparticles, and nanogels.

Liposomes and micelles are biodegradable NPs that share the characteristic of having a hollow core region, which is also known as a nanocapsule [17,18]. These specific features make them good candidates as drug matrices. Micelles are the most-used release systems for water-insoluble therapeutic agents. The hydrophilic exterior protects them from physiological processes, while the hydrophobic core can contain chemicals that are water insoluble, making them versatile delivery systems for active compounds [19].

**Figure 1:**
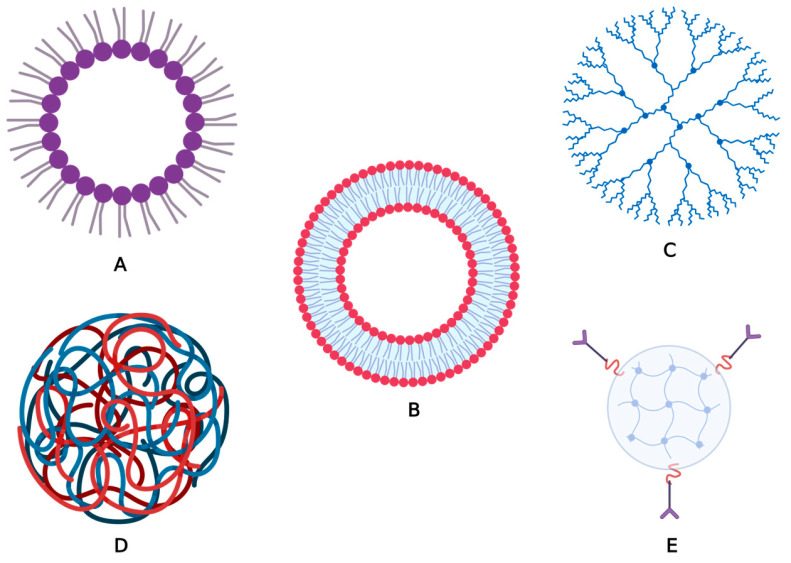
Most well-known organic NPs: (**A**) mycelium; (**B**) liposome; (**C**) dendrimer; (**D**) polymeric nanoparticle; (**E**) nanogel.

Liposomes are synthesized vesicles, that show great flexibility and can be conjugated with different lipid substances, thus having a wide variety of structures, sizes, and compositions. The most important advantage of liposomes is their ability to fuse with cell membranes, followed by the release of the active substance [20,21]. Multilamellar liposomes contain several lipid layers separated by hydrophilic layers, which makes them suitable for the encapsulation of both lipophilic and hydrophilic substances (Figure 1) [22]. Nowadays, liposomes are widely investigated for a variety of therapeutic applications, such as cancer diagnosis and therapy, targeted drug delivery, and antimicrobial therapy [21].

Dendrimers are extensively used for clinical applications due to their small size (1–5 nm) and structure, that allows them to be coupled with biocompatible compounds in order to decrease their cytotoxicity. They can act as vehicles for vaccines, genes, or drugs [23,24]. Despite their versatility, some have also shown a high risk of aggregation and toxicity, with only a few representatives being approved by the FDA (Food and Drug Administration) [10].

Polymeric NPs can be either synthetic or natural and can be classified as nanocapsules or nanospheres. These NPs can be synthesized so as to contain therapeutic agents in high concentrations that can be released in a targeted manner [25,26]. For instance, several studies focused on the production and practical applications of poly (methyl methacrylate) (PMMA) dispersions [27,28]. PMMA, a commonly used amorphous synthetic polymer, appears to be promising for biomedical applications due to its lack of toxicity, low cost, minimal inflammatory reactions on tissues and simple processability [29].

Furthermore, protein NPs can be formed through the self-assembly of protein polymers, which consist of isolated proteins derived from animals or plants, such as collagen, gelatin, albumin or elastin. Protein subunits are self-assembled into effective drug delivery vehicles with the help of polymer-based NPs via genetic engineering [30,31]. Abraxane^®^ is one example of an FDA-approved protein nanoparticle drug that enables the delivery of paclitaxel by albumin in different types of breast cancer [32]. Ontak, an engineered protein combining IL-2 and the diphtheria toxin nanoparticle formulation used to treat persistent or recurrent cutaneous T-cell lymphoma, is another such example [33].

Nanogels are defined by the International Union of Pure and Applied Chemistry as “the particle of gel of any shape with an equivalent diameter of approximately 1 to 100 nm” [34]. When compared to other nanocarrier systems, nanogels offer several benefits, including a reduced rate of premature drug release, the capacity to encapsulate therapeutic compounds inside a single formulation, and simple delivery via the parenteral or mucosal routes. Some of their most researched uses include the administration of nucleic acids, cytokines and vaccines, including nasal vaccines, which appear to be the most promising [35].

### 2.2. Inorganic Nanoparticles

**Figure 2:**
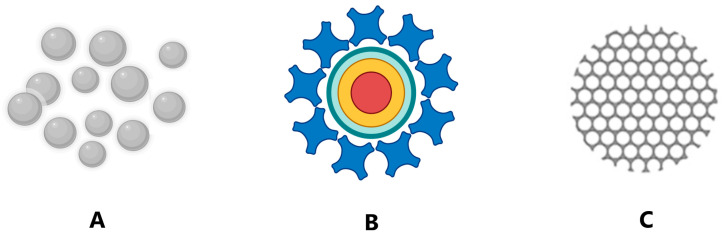
Most common inorganic nanoparticles: (**A**) metal NPs; (**B**) quantum dots, and (**C**) silica-based NPs.

Inorganic nanoparticles generally include metal and metal oxide NPs, quantum dots, and silica-based NPs (Figure 2).

Metal NPs can be synthesized using metals either by the top-down or bottom-up approaches [36]. Nearly all metals can be brought in the form of NPs [37]. The most used metals for the synthesis of NPs are gold (Au), silver (Ag), iron (Fe), aluminum (Al), cadmium (Cd), cobalt (Co), copper (Cu) and zinc (Zn) [16]. All NPs show distinctive general properties such as size, surface–volume ratio, pore size, electronic surface charge and surface density, crystalline or amorphous structure, spherical or cylindrical shape, and color, as well as reactivity and sensitivity to external factors (e.g., humidity, temperature, and light).

Metal oxide NPs are synthesized by modifying the properties of the corresponding metal. For instance, iron-based nanoparticles (FeNPs) spontaneously oxidize to iron oxide in the presence of oxygen, at room temperature, which generates an increase in reactivity compared to FeNPs. Most metal oxide NPs are manufactured using physical or chemical synthesis methods because they have the capacity to decrease in size when exposed to physical forces, such as those inside a rotating reactor, or due to their increased chemical reactivity [38]. Usually, aluminum oxide (Al_2_O_3_), iron oxide (Fe_2_O_3_), magnetite (Fe_3_O_4_), silicon dioxide (SiO_2_) and titanium oxide (TiO_2_) NPs are synthesized in this manner. These particles possess improved properties compared to those of the metals from which they originate [16,39].

Quantum dots are a certain type of nanoscale particle characterized by their small size and unique optical properties. They are made of semiconductor materials, typically cadmium selenide (CdSe), cadmium telluride (CdTe), or lead sulfide (PbS), and have diameters in the range of 2 to 10 nm. They are used for various applications, including displays, solar cells and bioimaging [40].

Silica-based NPs represent another type of inorganic nanomaterial composed of silicon and oxygen atoms in a crystalline configuration. Biocompatibility, chemical and thermal stability, as well as low toxicity make them promising materials for biomedical applications such as drug delivery, imaging, and biosensing [41].

Semimetal NPs also represent a compelling subgroup within the diverse domain of inorganic NPs. These nanomaterials possess intriguing electronic and optical properties, as well as potential therapeutic effects. Notable examples include selenium [42], antimony [43] and bismuth-based nanomaterials, that possess versatile biomedical applications [44].

Nonetheless, combining metal NPs with other nanostructures has shown remarkable potential for various specific medical applications. One notable example is the integration of gold NPs with silica nanoshells to create photothermal agents. These particles can selectively accumulate in tumor tissues, and when exposed to near-infrared light, they generate localized heat, effectively and specifically destroying cancer cells. This approach, known as photothermal therapy, holds promise in cancer treatment [45]. Gold NPs can also be encapsulated within liposomes, creating versatile drug delivery systems. These hybrid nanostructures enable the controlled release of therapeutic agents and can be guided to specific disease sites such as tumors, enhancing the effectiveness of chemotherapy while minimizing side effects [46,47]. Moreover, mesoporous silica-coated gold nanorods have been used as a theranostic nanoplatform for standard radiation diagnosis and therapy [48]. Similarly, incorporating silver NPs into hydrogel matrices can result in effective antimicrobial wound dressings. These wound dressings release silver ions over time, effectively preventing bacterial infections and promoting faster wound-healing [49].

### 2.3. Carbon-Based Nanoparticles

**Figure 3:**
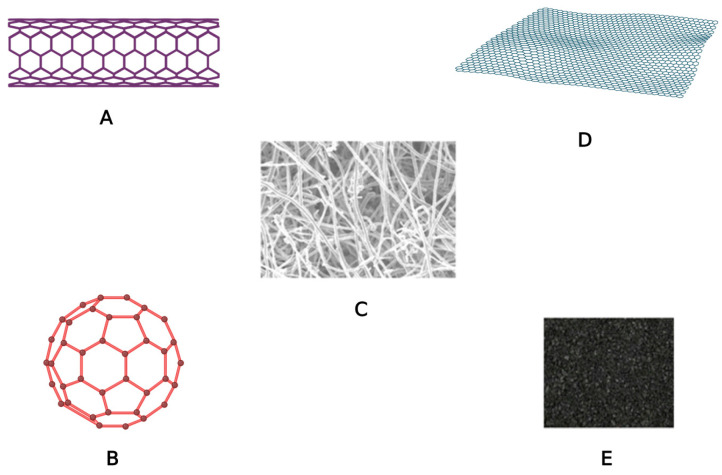
Carbon-based nanomaterials: (**A**) carbon nanotubes; (**B**) fullerenes; (**C**) carbon nanofibers; (**D**) graphene; (**E**) carbon black.

Carbon-based nanoparticles can be classified into fullerenes, graphene, carbon nanotubes, carbon nanofibers and carbon black (Figure 3). NPs that are made entirely of carbon are known as carbonaceous NPs [50].

Fullerenes are spherical molecules composed of up to 1500 carbon atoms connected by sp^2^ hybridization [51]. Studies have shown that fullerenes can be used to deliver antibiotics and antiviral agents, being also extensively employed in the development of high-performance MRI contrast agents, X-ray imaging contrast agents, photodynamic treatment, and gene delivery [52,53].

Graphene is an allotrope of carbon that consists of carbon atoms arranged in a hexagonal arrangement in a two-dimensional plane [54].

Carbon nanotubes are carbon-based tubular structures with a diameter of 1 nm and a length of 1–100 nm. Given that they can enter the cell by endocytosis or by direct insertion through the cell membrane, their size and shape make them desirable carriers for active substances [55]. In the case of carbon nanofibers, graphene sheets used to make nanotubes can be shaped like a cone or a cup, instead of a cylindrical shape [56].

Carbon black is an amorphous carbon material, generally spherical in shape, with a diameter ranging between 20 to 70 nm. The interaction between particles is so intense that they aggregate, thus forming agglomerates of around 500 nm [57].

## 3. Synthesis of Metal Nanoparticles

Metal NPs are generally obtained with the use of nanotechnology, by reducing the respective metal to its nuclear size. Their synthesis can be performed using several physical, chemical, and biological techniques.

Physical approaches prevent NPs from becoming contaminated with solvents but need a significant amount of energy for condensation and evaporation. Implicitly, the exceptionally high modulation of temperature and pressure also raises the cost of synthesis.

In chemical engineering, reducing and protective agents are used for the synthesis of NPs and agglomeration is prevented, thus generating NPs with high purity and stability. However, the contamination of synthetic NPs can be caused by a significant number of strong chemicals. 

**Figure 4:**
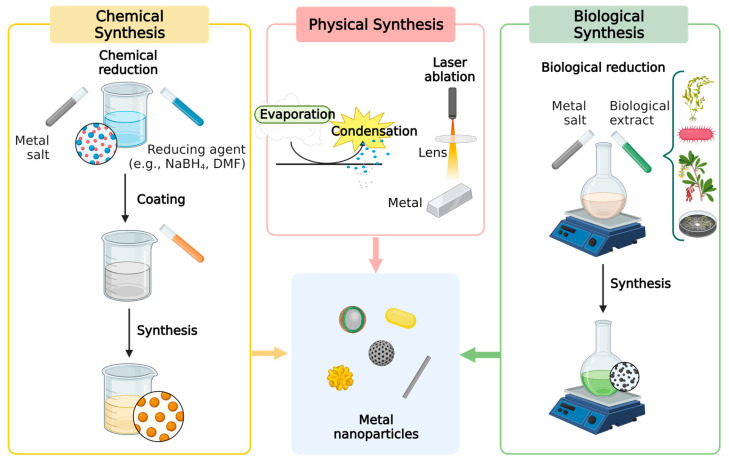
Common synthesis techniques employed for metal NPs.

The interest in the biological synthesis of NPs is based on the fact that it offers an eco-friendly and effective alternative to the chemical and physical methods. The biological technique employed in green synthesis varies depending on the type of reducing agent that is used, such as microorganisms (bacteria and fungi) and plants (Figure 4). 

One of the most cost-effective and environmentally friendly methods of synthesizing metal NPs is through the use of plants, which act as biofactories for NP- production. The potential scale-up of metal NPs obtained with the use of plants is immense due to their abundance, easy availability, and ability to be grown under diverse environmental conditions. Additionally, plant-based synthesis eliminates the need for expensive and hazardous chemicals typically used in conventional methods, making it a safer and more sustainable option. However, there are potential drawbacks associated with the use of plants for NP synthesis, such as variability in the size and shape of the produced NPs due to differences in plant species, plant-growth conditions, and harvesting times [58]. Nonetheless, considering the quantitative aspect, recent studies have reported yields of up to several grams of NPs per kilogram of plant material, making plant-based NP synthesis a viable option for industrial-scale production [59]. Consequently, synthesis using plant extracts is an interesting alternative for the large-scale manufacturing of NPs and has a great deal of potential for medical applications [60]. 

Considering the applied methods, the synthesis usually follows one of two well-known approaches: bottom-up or top-down.

The *bottom-up approach* is a constructive process that gradually leads to the synthesis of nanomaterials, starting from atoms to clusters, then to NPs. This method generally includes processes such as sol-gel, spinning and biosynthesis [61]. Other examples include sedimentation, reduction, green synthesis, centrifugation, biochemical synthesis, atomic layer deposition and molecular self-assembly [62].

On the other hand, the *top-down approach* seeks to reduce unprocessed, raw material to nanometric dimensions by destroying the initial structures using various physical forces to synthesize NPs [63]. Mechanical milling, nanolithography, laser ablation, sputtering, and thermal decomposition are some of the most well-known techniques that correspond to this approach [63].

Silver, gold, iron, cobalt, nickel, and some of their corresponding oxides (e.g., magnetite, cobalt ferrite) make up the majority of metal NPs employed in medical applications. They can be synthesized and modified with functional chemicals, which contain versatile groups that allow them to be combined with a variety of molecules, including therapeutic agents and biomolecules such as peptides, proteins, and DNA (deoxyribonucleic acid) [64]. For example, AuNPs have unique photothermal properties due to the free electrons on the surface that continually oscillate at a frequency dependent on their size and shape. Iron oxide NPs are among the majority of FDA-approved inorganic nanomedicines. Magnetic iron oxide NPs, composed of magnetite (Fe_3_O_4_) or maghemite (Fe_2_O_3_), possess superparamagnetic properties at certain sizes and have demonstrated efficacy as contrast agents, and drug delivery systems, for DNA extraction and thermal-based therapeutics [65].

### 3.1. Synthesis of Silver Nanoparticles (AgNPs)

Due to their exceptional physical and chemical properties, AgNPs are frequently used in numerous fields, such as medicine, agriculture, and different industries [66]. Such properties include optical, electrical, thermal, and high electrical conductivity, as well as biological properties [67,68,69]. Therefore, in order to meet the increasing demand for AgNPs, numerous synthesis techniques have been developed.

Both the *top-down* and *bottom-up* approaches demonstrate a high yield of synthesized AgNPs without the use of toxic chemicals that endanger human health and the environment. In the absence of capping agents, however, aggregation is frequently a challenging task. In addition, both methods consume more energy, require a longer synthesis time, and call for complex equipment, all of which increase operating expenses [70].

Typically, the evaporation–condensation method employs a gas-phase route that enables a tube furnace to produce nanospheres at atmospheric pressure. This technique has been used to synthesize nanospheres made from a variety of substances, including Au, Ag, and PbS [71]. At the center of the tube furnace, there is a vessel containing a source of base metal, which is evaporated into the carrier gas to enable the final synthesis. By modifying the design of the reaction facilities, the size, shape, and yield of NPs can be controlled. The tube furnace occupies a large area, has a high energy output, increases the surrounding temperature of the metal source, and requires more time to maintain thermal stability. In order to overcome these drawbacks, Jung et al. demonstrated that a ceramic heater can be effectively implemented in the synthesis of high concentration AgNPs [72].

Laser ablation is another technique applied in physical synthesis. Laser ablation of a bulk metal source in a liquid medium can be used to synthesize AgNPs. After irradiation with a pulsed laser, the medium contains only NPs of the source base metal, with no additional ions or compounds [73]. Therefore, in contrast to chemical synthesis, the synthesis of NPs by laser ablation is pure and contaminant-free, as this technique uses mild surfactants in the solvent and no other chemical reagents [74].

The advantages of physical methods include speed, the use of radiation as a reducing agent, and the lack of hazardous chemicals, whereas the disadvantages include low yield and high energy consumption, solvent contamination, and lack of uniform distribution [75,76,77].

Chemical synthesis methods have been commonly applied for metal NPs synthesis as a colloidal dispersion in an aqueous solution or organic solvent by reducing their metal salts [78]. For the synthesis of metal nanospheres, such as gold, silver, iron, zinc oxide, copper, palladium, platinum, various metal salts are used [79]. Reducing and capping agents can be easily modified in order to achieve the desired particles’ characteristics (e.g., size distribution, shape, dispersion rate) [80].

AgNPs are chemically synthesized mainly by Brust–Schiffrin synthesis (BSS) or the Turkevich method [74,81,82]. The chemical reduction of silver metal salts can be achieved using various reducing agents such as glucose, hydrazine, ascorbate, dimethylformamide, hydrogen, dextrose (Turkevich method) or sodium borohydride (BSS method) [83,84].

The chemical synthesis process typically uses three main components: metal precursors, reducing agents and stabilizing or coating agents. Essentially, the reduction of silver salts involves a nucleation in two steps and a subsequent growth. The main advantage of chemical methods is their high yield, in contrast to the low yield of physical methods. Nonetheless, these methods are extremely expensive, and the materials used for AgNPs synthesis, such as citrate, borohydride, thio-glycerol and 2-mercaptoethanol are toxic and unsafe [85]. Apart from these disadvantages, the obtained particles lack the desired purity, as numerous chemicals have been detected on their surfaces. Additionally, it is quite difficult to prepare AgNPs with a well-defined size, as this would require an additional step to prevent particle aggregation [86].

In order to overcome the limitations of chemical methods, viable biological alternatives have emerged. Recently, it has been demonstrated that biologically mediated synthesis is a simple, cost-effective, reliable, and environmentally friendly method. Moreover, much attention has been paid to the high-yield production of size-defined AgNPs using various biological systems such as bacteria, fungi, plant extracts or even small biomolecules (e.g., vitamins, amino acids) as an alternative to chemical methods, not only for AgNPs, but also for the synthesis of several other types of NPs [87,88,89,90]. The biosynthesized NPs’ yield is a pivotal metric, often ranging from 60% to 90% or higher. Achieving a high yield is essential for the economic viability and scalability of nanoparticle production. Therefore, researchers diligently optimize parameters such as temperature, pH, and precursor concentrations to enhance production [59]. Selecting appropriate plant extracts or sustainable materials is equally crucial. A high yield in green synthesis not only ensures efficient production but also supports the sustainability and applicability of these nanomaterials across various domains, ranging from medicine to catalysis and environmental remediation [91].

Biological synthesis depends mostly upon three elements: the solvent, the reducing agent, and the non-toxic material. The main advantage of biological methods is the presence of amino acids, proteins, and secondary metabolites in the synthesis process, which eliminates the need for an additional step so as to prevent particle aggregation. Furthermore, such techniques appear to provide a controlled particle size and shape, which is crucial for a variety of biomedical applications [87]. Given that nowadays the focus is set on biosynthesized nanomaterials, some examples of biological reducing agents used for obtaining silver and gold NPs, respectively, and the corresponding optimal conditions, can be found in Table 1.

Nevertheless, the presence of some biomaterials in the extract could raise issues during the production of metal NPs, including delayed reaction kinetics that could further result in low stability. These concerns are usually counteracted by plant polyphenols [92]. Therefore, in order to overcome such potential limitations, recent studies have also focused on fabricating particles with the help of isolated biocompounds, such as curcumin, kaempferol, apigenin and quercetin [93,94].

**Table 1:** Examples of biological agents and conditions used for AgNPs/AuNPs synthesis.

NPs Type	Plants				
**AgNPs**	**Species**	**Biological material,** **conditions**	**Salt ** **concentration** **(AgNO_3_)**	**Mixing conditions**	**Reference**
	*Adenia trilobata*	leaves, 5 g % (70 °C for 25 min)	1–5 mM	60 °C, 30 min and kept at room temperature for 1 h	[95]
	*Buchanania lanzan*	leaves, 5 g % (boiled for 5 min)	0.5 mM	70 °C, 1 h	[96]
	*Cannabis sativa*	leaves, 1 g % (60 °C)	3 mM	37 °C, 24 h	[97]
	*Embelia ribes*	fruits, extraction of embelin	1 mM	30 min in the dark and incubation for 24 h	[98]
	*Lythrum salicaria*	aerial part, 10 g % (40 °C)	3 mM	60 °C, pH = 8, 180 min	[99]
	*Moringa oleifera*	leaves, 10 g % (100 °C for 20–30 min)	1 mM	60–80 °C, 60 min	[100]
	*Picea abies,* *Pinus nigra*	bark, 10 g % (60 °C)	1 mM	room temperature, 60 min	[101]
	*Sanvitalia procumbens*	plant, 10 g % (70 °C)	0.01 M	70 °C, pH = 8, 8 h	[102]
	*Tagetes erecta*	flowers, 10 g %	3 mM	40 °C, pH = 8, 75 min	[103]
	*Theobroma cacao*	cocoa pods and leaves, 15 g % (boiled for 10 min)	1 mM	room temperature, 4–24 h	[104]
	**Microorganisms**				
	*Bacillus cereus*	37 °C, 24 h, 120 rpm	1 mM	48.5 °C, pH = 9, 69 h	[105]
	*Bacillus subtilis,* *B. cereus,* *B. megaterium,* *B. pumilus,* *B. circulans*	isolates grown aerobically, 37 °C, 24 h	1 mM	30 °C, 24 h	[106]
	*Klebsiella pneumonia*	37 °C, 24 h	4 mM	37 °C, pH = 10, 24 h	[107]
	*Lactobacillus bulgaricus*	isolated colonies from raw milk, 37 °C	1 mM	room temperature	[108]
	*Aspergillus terreus*	strain isolated from soil, 25 °C, 5 days, 200 rpm	1 mM	25 °C, 200 rpm	[109]
	*Penicillium verrucosum*	strain isolated from vegetable-cultivated greenhouse soil, 25 °C, 7 days, 150 rpm	10 mM	25 °C, in the dark	[110]
	**Algae**				
	*Chlorella vulgaris*	algal suspension	10 mM	25 °C, 48 h	[111]
	*Dunaliella salina*	culture grown for 2 weeks, 12 h in light and 12 h in dark	1 mM	in the dark, 48 h	[112]
	*Ulva rigida* (green alga) *Cystoseira myrica* (brown alga) *Gracilaria foliifera* (red alga)	ethanolic and aqueous extracts obtained from dried algal powder, room temperature, 24 h	1 mM	70 °C, 15 min	[113]
**AuNPs**	**Species**	**Biological material, conditions**	**Salt concentration** **(HAuCl_4_)**	**Mixing conditions**	**Reference**
	**Plants**				
	*Allium ampeloprasum*	leaves, 10 g % (boiled)	1 mM	room temperature, 30 min	[114]
	*Annona muricata*	leaves, ~ 13 g % (20 g–150 mL) (boiled 20 min)	1 mM	room temperature, 22 h	[115]
	*Citrus maxima*	peel, 1 g % (boiled for 10 min)	100 mM	room temperature, 1 h	[116]
	*Dillenia indica*	leaves, 1 g % (100 °C for 30 min)	1 mM	dark, 30 min	[117]
	*Dracocephalum kotschyi*	leaves, ~ 7 g % (5 g–75 mL) boiled	1 mM	10 min	[118]
	*Lonicera japonica*	flowers, 10 g % (boiled 20 min)	1 mM	70 °C, 30 min	[119]
	*Mentha* and *Pelargonium*	plant (boiled 20 min)	-	boiling	[120]
	*Pergularia daemia*	leaves, 10 g % (boiling at 60 °C for 30 min)	1 mM	15 min	[121]
	*Platycodon grandiflorum*	leaves, 10 g % (boiled for 20 min)	1 mM	50 °C, 15 min	[122]
	*Tecoma capensis*	leaves, 5 g % (heated till boiling ~20 min)	0.011 M	heating, 10 min	[123]
	**Microorganisms**				
	*Bacillus cereus* *Fusarium oxysporum*	bacterial culture incubated at 37 °C, 150 rpm, 24 h fungal culture incubated at 30 °C, 150 rpm, 72 h	1 mM	37 °C, 200 rpm, 24 h	[124]
	*Pseudomonas*	culture, 24 h	1 mM	80–85 °C, 30 min	[125]
	*Candida albicans*	cytosolic extract	1 mM	24 h	[126]
	**Algae**				
	*Chlorella sorokiniana*	aqueous extract, 80 °C, 20 min	1 mM	80 °C, pH = 8, 60 min	[127]
	*Galaxaura elongata*	powder form and ethanolic extract	1 mM	room temperature, 120 rpm, 12 h	[128]
	*Oscillatoria sp.* *Spirulina platensis*	microalgal cultures	5 mM	room temperature, 24 h	[129]

Current research highlights the benefits that the use of plant material (plants, microorganisms, algae) confers in obtaining NPs. However, the limitations they may have must also be taken into account. Table 2 summarizes these advantages and disadvantages.

### 3.2. Synthesis of Gold Nanoparticles (AuNPs)

The synthesis of AuNPs generally adheres to the two above-mentioned methods.

Physical methods, including pyrolysis, nanolithography, thermolysis, and radiation-induced methods involve the controlled processes of cutting, milling, and modeling the materials into the desired order and shape. The high cost of the process, which requires a massive amount of energy to maintain the high pressure and high temperature conditions, is, however, a limitation of these methods [139,140,141].

In the second approach, AuNPs are created through the self-assembly of individual species (atoms, molecules, or clusters) using chemical or biological processes. This method is less expensive and provides greater control over the final development of a product with a more uniform size, shape, and chemical composition [142].

The chemical synthesis of AuNPs involves the reduction of a gold salt and subsequent stabilization of NPs. Turkevich and Brust–Schiffrin are the two conventional chemical techniques for obtaining AuNPs. Turkevich described a simple method for producing spherical AuNPs by reducing hydrogentetrachloroaurate(III) (HAuCl_4_) with trisodium citrate, which acts as both a reducing agent and a stabilizer [143]. The discovery of the Brust–Schiffrin method allowed a simple approach to synthesize stable AuNPs of controlled size. Using tetraoctylammonium bromide as a phase transfer agent, AuCl_4_^-^ was transferred to toluene from an aqueous phase and reduced, in the presence of dodecanethiol, with NaBH_4_. The addition of the reducing agent caused the organic phase to change color from orange to dark brown, which unequivocally demonstrated the formation of AuNPs [82].

Although chemical methods are the most common way to synthesize metal nanomaterials, the use of expensive and toxic reagents as reducing and stabilizing agents restricts their applications. Additionally, such NPs may also have negative effects when used for biomedical applications [144,145]. Consequently, there is a growing need to develop environmentally friendly and cost-effective nanoparticle synthesis techniques that do not employ toxic substances. Therefore, in recent years, biological synthesis has gained significant attention as a green and environmentally friendly approach, that uses microorganisms, enzymes, and plants to produce NPs [146,147].

## 4. Characterization of Metal NPs

**Figure 5:**
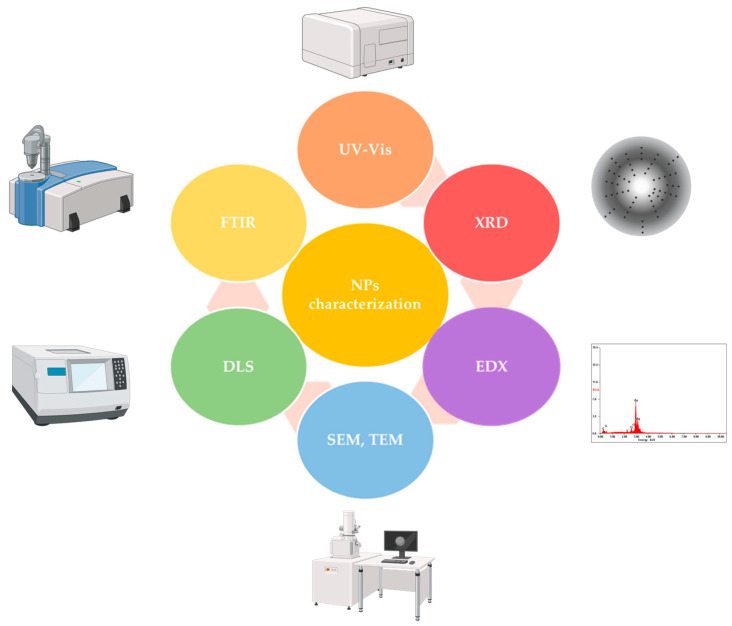
The most used characterization techniques: UV-Vis—UV-Vis Spectroscopy; XRD—X-Ray Diffraction; EDX—Energy Dispersive X-Ray Analysis; SEM—Scanning Electron Microscopy, TEM—Transmission Electron Microscopy; DLS—Dynamic Light Scattering; FTIR—Fourier-Transform Infrared Spectroscopy.

The appropriate characterization of metal NPs is essential for obtaining in-depth information that will influence their application in various fields, as well as for assessing their toxicity [148]. Figure 5 depicts the variety of techniques used to characterize metal NPs, such as UV-Vis spectroscopy, SEM (Scanning Electron Microscopy), TEM (Transmission Electron Microscopy), FTIR (Fourier-Transform Infrared Spectroscopy) and EDX (Energy Dispersive X-Ray) analysis, techniques that can reveal their composition, morphology, and size [149].

### 4.1. Visual Inspection and UV-Vis Spectroscopy

For the initial observation of NPs formation, the color change of the extract-metal salt mixture can be observed. The color varies from the initial color of the extract to orange-brown (AgNPs obtained using *Eucalyptus camaldulensis*) or dark brown (AgNPs obtained using *Prunus persica*) in the case of AgNPs, whereas for AuNPs, the color indicating the obtaining of NPs can be pink (AuNPs from Tamarindus indica) or ruby-red (AuNPs from *Mentha piperita*, *Melissa officinalis* and *Salvia officinalis*) [150,151,152,153].

Using UV-is spectroscopy, the formation of AgNPs (by reducing Ag^+^ to Ag^0^) and AuNPs (by reducing Au^3+^ to Au^0^) with the help of biomolecules present in plant extracts is demonstrated by the appearance of SPR (Surface Plasmon Resonance) that is highlighted in the UV-Vis spectra by a maximum absorbance around specific wavelengths. In general, the maximum absorption of AgNPs occurs between 400 and 500 nm, while that of AuNPs occurs between 500 and 600 nm. For instance, in the case of AgNPs synthesized from *Gleichenia pectinata* (obtained using a 10 g % methanolic extract, 5 mM silver nitrate and 1:5 extract–silver nitrate volume ratio), the SPR band appeared at 460 nm [154]. However, AgNPs obtained from *Euterpe oleracea*, *Oenocarpus bacaba* and *Mauritia flexuosa* (obtained using a 100 mg/mL aqueous extract, 1 mmol/L silver nitrate and incubation under low light conditions at 75 °C for 90 min), the SPR band could be found in the 420–430 nm range [155].

For AuNPs obtained from *Dodonaea viscose* leaves (in the presence of 1000 ppm tetrachloroaurate), the SPR band appears at 600 nm [156], and around 530–540 nm for those obtained from green tea and *Juniperus communis* extract [157]. Another example shows the characteristic peak for AuNPs derived from *Platycodon grandiflorum* extract (with 1 mM HAuCl_4_ solution) at 545 nm [122].

In addition, the nature of the colloidal dispersion and the shape of AgNPs can be deduced using the data obtained during UV-Vis analysis. For example, according to Mie’s theory, the presence of a single resonance band corresponds to spherical particles [158]. Moreover, according to Miskovská et al., a peak absorption around 420 nm indicates monodisperse and smaller AgNPs, whereas a peak at 440 nm indicates polydisperse AgNPs [159]. Other studies also confirm the relationship between NPs size and shape and the increase or decrease of wavelength, through the observation of different red or blue shifts [160,161]. Additionally, for AuNPs, smaller particles are typically associated with shorter wavelengths [162].

### 4.2. FTIR Spectroscopy

FTIR analysis can be used to highlight functional groups of different active substances or of plant secondary metabolites that are responsible for the reduction, capping, and stabilization of both AgNPs and AuNPs. Absorption bands around 3300–3400 cm^−1^, 2940 cm^−1^, 1600–1700 cm^−^^1^, 1350–1450 cm^−^^1^ and 1000–1100 cm^−^^1^ correspond to O-H stretching and C-H stretching of aromatic compounds, C=O stretching of carbonyl compounds, to C-C, C-O-C, C-N, C-O bonds, N-H stretch vibration of amides, and to C=C groups. Such bonds can be easily found in phytochemicals such as polyphenols, proteins, alcohols, terpenoids, enzymes and alkaloids [154,155].

When comparing the FTIR spectra of nanoparticles to those of extracts, shifted or diminished peaks are revealed, which can be explained by the phytochemicals’ participation in the synthesis and capping processes [163]. Moreover, the literature describes a new band around 640–670 cm^−^^1^ for AuNPs, that can be attributed to metal formation [153,156].

### 4.3. Dynamic Light Scattering (DLS) and Zeta Potential Analysis

The surface charge of particles determines the interactions with the targeted site. In order to measure the electric charge of the surface and the electrostatic stability of the dispersion in the liquid phase, the zeta potentiometer is used [164].

Due to the ionizable or adsorbed groups on the surface, the zeta potential indicates the surface charge of particles. As a result, it is possible to study the aggregation tendency and understand the stability of NPs in suspension. A zeta potential value greater than +30 mV or less than −30 mV determines the production of a very stable nanoparticle dispersion [165]. A zeta potential closer to 0 mV suggests a larger probability of aggregation [155]. Generally, the zeta potential has a negative value, which implies electrostatic repulsion between particles, thus avoiding agglomeration and flocculation and ensuring stability.

The PdI (polydispersity index) represents the width of particle size distribution. The PdI range is between zero and one, with zero suggesting a monodisperse distribution and uniform size and one indicating a polydisperse distribution with various nanoparticle sizes [166]. 

DLS is used to determine the hydrodynamic diameter of NPs in a medium (by measuring dynamic fluctuations of light-scattering intensity due to the Brownian motion of particles). For instance, there are differences between NPs as they can be coated with plant extract biomolecules that have varying molecular weights [167].

### 4.4. SEM, TEM, EDX

The elemental composition can be determined using EDX analysis. Strong absorption peaks at 3 keV and 2 keV indicate the binding energies of silver and gold, respectively. In the case of biosynthesized NPs, additional elements that may be present, such as carbon, oxygen, aluminum, and potassium, are a result of plant phytochemicals that act as capping elements on the surface of AgNPs [154,163,168].

The selection of NPs for various applications is influenced significantly by the variety of forms and surface conformations that they exhibit. The surface morphology can be crystalline or amorphous, smooth, or rough, and geometries range from spherical, flat, cylindrical, tubular, or conical to irregular. Electron microscopy techniques, such as SEM or TEM, are used to analyze the surface morphology [169]. For example, AgNPs obtained from *M. oleifera* leaves extract (1 mM AgNO_3,_ 60–80 °C, 1 h) showed spherical nanoparticles, with a particle size in the 10–25 nm range [100]. On the other hand, for AuNPs synthesized from *Citrullus colocynthis* seed extract (1 mM chloroauric acid, extract–salt 1:2 volume ratio, 70 °C, 20–30 min), the obtained particles presented spherical shape and a particle size in the 7–33 nm range [170].

Moreover, the size, morphology and shape of NPs can be determined by TEM analysis. In the case of AgNPs synthesized from *Euterpe oleracea*, *Oenocarpus bacaba* and *Mauritia flexuosa* leaves extract, TEM analysis revealed polydisperse AgNPs, mostly spherical in shape, without the formation of large agglomerates [155]. Another given example is that of AgNPs obtained from parts of *Crataegus ambigua*, with TEM analysis demonstrating the obtaining of particles with an average size of 32 nm, that are spherical in shape [171].

In the case of AuNPs, the shape may be more variable. For example, AuNPs obtained from green tea have an hexagonal shape, those from *Juniperus communis* extract are circular and hexagonal, while those from green coconut water are circular, triangular, hexagonal and rod-shaped [157]. AuNPs from *Salvia officinalis* extract (obtained using 200 mg/l HAuCl_4_) were spherical, triangular, star-shaped, and rod-shaped, whereas AuNPs from *Melissa officinalis* extract were triangular, star- and rod-shaped [153]. Botteon et al. correlated different geometries of AuNPs obtained from Brazilian red propolis with their size and concluded that particles with triangle, pentagon, hexagon and rod shapes have large sizes, while those with spherical shapes are usually smaller [172]. This can be explained by the amount of phytochemicals that coat and stabilize the surface of nanoparticles, which determines their anisotropic nature [173].

SEM and TEM can reveal the coating of biosynthesized NPs, which is generally due to plant phytochemicals that cover the particles’ surface [99,155,174]. Verma and Mehata demonstrated that NPs size is influenced by synthesis conditions. Thus, for AgNPs synthesized from *Azadirachta indica*, an increase in the extract concentration and pH results in an increase in particle size, whereas an increase in temperature results in a decrease in size [175]. Additionally, Bhaskaran et al. demonstrated that the gold salt concentration influences the size and form of AuNPs generated from *Medicago sativa* cell suspension cultures. If, at a concentration of 10 ppm gold salt, the majority of the formed NPs are spherical, the percentage of AuNPs with other shapes, such as hexagons, pentagons, and triangles, will grow as the salt concentration rises. Increasing the concentration of the gold salt will also simultaneously increases the size of particles [176].

### 4.5. XRD Analysis

The XRD technique analyzes the nature of nanoparticles. According to Bragg’s model of diffraction given by the Joint Committee on Powder Diffraction Standards—International Center for Diffraction, sharp and clear peaks (2Θ) of particles are located around 38, 44, 64º which can be assigned to (101), (111), (200) and (220) planes that indicate a high degree of crystallinity of AgNPs (JCPDS No 65-2871) [154,177]. 

For AuNPs, the pattern shows peaks around 38, 44, 64 and 77°, with corresponding lattice plane at (111), (200), (220) and (311) of face-centered cubic crystal structure (JCPDS No. 01-089-3697) [153].

The Scherrer equation, originally formulated by Paul Scherrer in 1918, has remained a cornerstone in X-ray crystallography and materials science. When applied to the XRD analysis of NPs, it provides critical insights into their structural characteristics, size and crystalline quality. This equation is particularly relevant in the study of metal NPs because their properties are often closely linked to their size and structure [178].

One important aspect to note is that the broadening of XRD peaks is primarily influenced by the finite size of the crystalline domains in the sample. Larger nanocrystals will exhibit narrower peaks, while smaller ones will result in broader peaks. This relationship between crystallite size and peak-broadening represents the very basis of the Scherrer equation. Moreover, the equation can be used to estimate the overall size distribution within a sample by analyzing multiple diffraction peaks. Implicitly, the Scherrer equation provides an average size, and in actual samples, size distributions are often present due to variations in the synthesis process [179].

### 4.6. Other Characterization Techniques

Other techniques such as inductively coupled plasma-mass spectrometry and capillary electrophoresis can be used for detection or for the study of other NPs characteristics, such as mass distribution, electrical conductivity or resistance [180,181]. The applied techniques are different depending on the complexity of the matrix, and the concentration of the analyte, as well as on the physicochemical characteristics. In certain cases, sample dilution may be required additionally so as to accurately detect the concentration of the analyte. The characteristics that can be established using some of the previously mentioned techniques are summarized below.

Particle size represents one of the fundamental characteristics of NPs and particles can be classified as nano or micro. Usually, the particle size and distribution can be determined by electron microscopy. The images obtained by SEM or TEM are employed in order to determine the size of particles and clusters, while laser diffraction techniques are used to measure the raw material in the solid phase [182].

The surface area is another essential characteristic of nanomaterials. In describing the properties and performances of such particles, the area-to-volume ratio is of critical importance. Typically, the surface area is determined using Brunauer–Emmett–Teller (BET) analysis. In a liquid phase, conventional titration can be used to analyze the surface of NPs, but the process is laborious and time-consuming; therefore, NMR is usually preferred. The differential mobility analyzer is used to measure the NPs’ gas-phase surface area [183].

The purity and effectiveness of NPs are determined by their chemical or elemental composition. Large concentrations of secondary or undesirable components might impair performance or potentially cause adverse effects, and chemicals could contaminate the final product. Besides FTIR spectroscopy, X-ray photoelectron spectroscopy can also be used to analyze the composition [184]. 

In other cases, the particles initially undergo a process of chemical digestion before being submitted to wet chemical examination in specific techniques such as chromatography, atomic emission spectroscopy, and mass spectroscopy. The particles detected in the gas phase are collected using filtration or electrostatic collection, and then they are subjected to spectrometric or wet chromatographic examination [17].

The experimental science of crystallography examines the configuration of atoms and molecules in crystalline materials. To emphasize the structural arrangement, X-ray, electron, or neutron diffraction methods are typically used in the crystallography of NPs [185].

Testing the stability of NPs is also crucial to ensure their performance and safety in various applications. Nanoparticle stability can be assessed through a combination of physical, chemical, and biological methods, ranging from visual inspection to biological compatibility assessment. However, one of the most common methods is represented by UV-Vis spectroscopy that can monitor changes in the absorption spectrum of the NPs [186]. Aggregation or precipitation often results in a shift or broadening of the absorption peaks [187].

## 5. Biomedical Applications of Metal NPs

**Figure 6:**
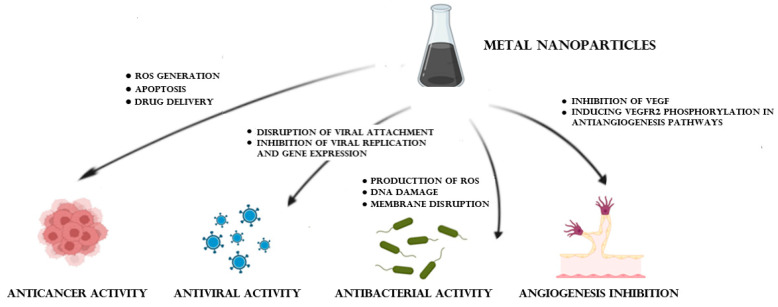
Biological activities of metal NPs and their potential mechanisms (VEGF—vascular endothelial growth factor; VEGFR—vascular endothelial growth factor receptor).

Metal NPs have gained significant attention due to their unique physical, chemical, and optical properties. These nanoscale metal structures have demonstrated a wide range of applications in various medical areas. Figure 6 schematically shows the most promising biomedical uses of metal NPs and the mechanisms involved.

One of the major applications is in targeted drug delivery, where metal particles can be functionalized with specific ligands or antibodies to selectively target and deliver therapeutic agents to cells or tissues. Additionally, metal NPs have shown promise in medical imaging as contrast agents, enhancing the visualization of tissues and enabling early disease detection. They also find utility in biosensing and diagnostic applications, where their optical properties facilitate sensitive and rapid detection of biomarkers. Furthermore, metal NPs have been explored for their antimicrobial properties, with potential applications in combating bacterial infections and developing novel antimicrobial agents. 

### 5.1. Diagnostics

Some nanoparticles are generally rendered as stable and biocompatible, but they also have special qualities, including magnetic properties. As a result, an externally generated magnetic field can be used to direct magnetic NPs to a specific position inside the body. Regarding their application in medicine, a crucial parameter is their magnetic susceptibility, which is defined by the relationship between the applied field and the induced magnetization. For instance, super-paramagnetic iron oxide nanoparticles (SPIONs) are frequently used in clinics as contrast agents for magnetic resonance imaging (MRI) [188].

Additionally, it has been observed that, when administered intravenously, iron oxide NPs with diameters ranging from 50 to 150 nm are taken up by macrophages and accumulate in the liver, spleen, lymph nodes and bone marrow. This could prove useful for generating contrast in several diagnostic techniques [189]. In this regard, Ferrucci, Marchal and Deng used this feature to visualize the primary lesions of the liver in cases of hepatocellular carcinoma and liver metastases [190,191,192]. In contrast to the healthy liver tissue, tumor lesions are characterized by a lower uptake of SPIONs in the macrophages compared to the healthy liver parenchyma. Santoro et al. also used SPIONs to distinguish malignant from benign neoplastic liver lesions [193].

Metal NPs with diameters between 20–30 nm or less, called ultra-small superparamagnetic iron oxides (USPIO), have a long circulation time and have been used in lymphography primarily because, after intravenous administration, they extravasate into tissues, where they are picked up by the lymphatic circulation and reach the macrophages in the lymph nodes. Lymph node staging, which is essential for both classifying cancer and determining the best treatment plan, can be carried out using MRI [194]. Nonetheless, USPIO compounds could also serve for imaging parotid and other salivary gland tumors [195].

The ability of metal NPs to accumulate in macrophages can also be used to gather data on the development and stage of cardiovascular diseases such as atherosclerosis. Macrophages can be loaded with iron oxide NPs and viewed using MRI in order to identify inflammatory lesions [196,197]. In rabbits with susceptible or ruptured atherosclerotic plaques, Kaneko et al. noticed a rise in the absorption of USPIO/SPION [198]. Similar characteristics were also observed in humans [199]. Moreover, in the diagnosis and staging of multiple sclerosis, MRI has been used to assess the integrity of the blood-brain barrier and to localize inflammatory lesions in the brain. Tourdias et al. demonstrated that, by combining gadolinium with iron NPs, a much greater precision was achieved in the detection of such lesions [200].

The development of new AuNP-based contrast agents for MRI is rapidly evolving, and in addition to gadolinium chelates, several new and highly effective contrast agents have been reported [201]. For instance, gadolinium chelates now used for clinical diagnosis can be transported via AuNPs to increase the sensitivity of MRI [202].

Given the variety of processes used to control their size, shape, and distribution, AuNPs represent promising substrates for building prototypes for diagnosis and treatment. In addition, the organic coating that stabilizes the metal surface can be easily modified so as to control the interaction of NPs with biological systems [203].

Taking into account their optical and magnetic properties, AuNPs, as well as AgNPs, have also been intensively used in the diagnosis and bioimaging of cancer cells. Several studies focused on the potential use of the magnetic properties of AgNPs in detecting cancer cells [204]. Moreover, other researchers have developed a gold nanoparticle-based sensor capable of detecting lung cancer by analyzing the patient’s breath [205]. A more recent clinical study on AuNPs showed that such materials can be used for the screening of gastrointestinal tract tumors [206].

Other potential uses of silver and gold NPs focus on the detection of certain viruses. One example is represented by the detection of human immunodeficiency virus-1 (HIV-1) protease using electrochemical impedance spectroscopy [207]. AuNPs conjugated with hepatitis B virus (HBV) DNA can be used to directly detect the presence of HBV DNA using fluorescence-based methods, which are simple and low-cost [208]. Moreover, Yen et al. have successfully investigated AgNPs conjugated with antibodies for the detection of yellow fever and Dengue virus proteins and the Zaire ebolavirus glycoprotein [209].

Using a colorimetric method, Balakumar et al. also used AgNPs for the recognition and detection of lead, which could represent a possible application in the diagnosis of saturnism [210]. The use of AuNPs for the detection of *Mycobacterium tuberculosis*, the etiological agent that causes tuberculosis, also employing colorimetry, was another successful application of such particles. This type of approach also aided defining diabetes as a complex disease [211].

Furthermore, AuNPs were used to determine the concentration of amyloid-derivative-B-ligand, a potential marker for Alzheimer’s disease (AD), in the cerebrospinal fluid of affected individuals [212]. AuNPs optical biosensors were also used to monitor the interaction between the antigen (amyloid β-derived diffusible ligands) and its specific antibodies, with the aim of characterizing AD [213]. Interestingly, a recent study showed that AuNPs might also play an important role not only in the diagnosis of AD, but also in its treatment, by preventing neuroinflammation and impaired cognition on an okadaic acid-induced model in rats [214]. Calorimetric biosensors based on gold NPs also have rather promising detection limits for proteins and nucleic acids [215]. Nonetheless, several new biotechnological methods based on the expression of specific peptides on the surface of phages have been developed to improve the detection limit [216,217].

### 5.2. Anticancer Activity

NPs are used as vectors for diagnostic, hydrophobic medicine, therapeutics and especially in the delivery of antineoplastic agents to cancerous tissues, where NPs can penetrate and deliver the drug to a specific targeted site [218]. NPs are also attracting significant interest as carriers for the applications in drug and gene delivery, or as biosensors, where a direct contact with blood occurs [219]. Jagtap et al. investigated the in vitro anticancer potential of embelin fabricated AgNPs, the results confirming a promising anticancer effect specifically against A549 lung cancer cells [98].

Metal NPs are more cytotoxic to cancerous cell lines than to normal cells. The cytotoxicity of metal NPs has been explained by a number of different mechanisms, including the production of reactive oxygen species (ROS), permeabilization of the mitochondrial outer membrane, and specific DNA cleavage, all of which result in the death of the cancer cell via apoptosis, autophagy, and necrosis [220].

Many metal NPs are used in medicine, particularly in the treatment and diagnosis of cancer, considering optical and localized surface plasmon resonance, as well as their rather low cytotoxicity. Due to their characteristics, the photothermal conversion and heating of the targeted tumor tissue happen when the right wavelength of light is applied as an external stimulus, thus killing cancer cells. Iron oxide NPs can be used in this anticancer procedure as well, when exposed to an alternating external magnetic field that causes particle movement and local heating. This hyperthermic effect that has been employed in tumor therapy can cause tissue damage in the proximity of NPs [221].

The use of magnetic drug targeting to improve SPION accumulation in pathological regions has been investigated. This implies the existence of a strong external magnetic field gradient that guides SPIONs to a specific target region, such as a tumor, where an alternating magnetic field can produce hyperthermia or inducible drug release [222].

Amatya et al. studied the photothermal activity of iron oxide NPs modified with polyethylene glycol (PEG) and starch in order to prevent aggregation and improve biocompatibility. The modified particles were injected intravenously into U87 MG xenograft tumor-bearing mice (human cerebral glioblastoma) and were used as agents to treat cancer at a wavelength of 885 nm (NIR domain). The tumor site was irradiated with a laser, and the modified nanoparticles demonstrated the most prominent reduction in tumor size compared to control groups [223]. Moreover, Ge and collaborators proposed the encapsulation and transport of medicinal substances in iron oxide NPs to a magnetically targeted site. Using complementary photothermal therapy in conjunction with laser irradiation could lead to both a direct and indirect (via immune activation) means of significantly reducing the tumor’s volume [224].

Metal NPs also hold immense promise in the fields of radiotherapy and electric field-induced hyperthermia. In radiotherapy, NPs can be precisely delivered to tumor sites, enhancing the therapeutic effect while minimizing damage to healthy tissue [225]. By loading particles with radiation-absorbing materials, such as gold or hafnium oxide, they become radiation amplifiers, intensifying their tumor-killing capacity [226]. Additionally, when exposed to external electromagnetic fields, the particles generate localized heat, ideal for electric field-induced hyperthermia. This controlled heating can selectively damage cancer cells, improving treatment outcomes. The integration of NPs into these therapies represents a futuristic approach to cancer treatment, offering a potent combination of precision and potency in the fight against this disease [227].

Moreover, metal NPs have been used as intracellular carriers of nano-drugs into specific cells using various methods such as mechanical bombardment or electroporation [228]. Electroporation is one of the most successfully applied methods, given its improved transfer efficiency. For instance, the combination of functionalized gold NPs and electroporation can lead to the improvement of radiotherapy efficacy in cancers associated with human papillomavirus [229].

AuNPs are used for drug delivery, where light irradiation can trigger the drug’s release at the targeted site [230]. As previously mentioned, different ligands, such as peptides, proteins, or DNA, can optimize the surface of such particles. Moreover, in vivo studies of AuNPs demonstrated that they readily diffuse into tumor cells and therefore are highly effective in tumor targeting [231]. One proposed treatment involves targeted chemotherapy that delivers a tumor-killing agent, TNFα, to malignant tumors. CYT-6901 represents a first in nanomedicine, consisting of AuNPs coated with TNFα factor. Goel R et al. performed an in vivo study of this drug in human prostate tumor-bearing mice and the results indicated the specific accumulation of TNFα in tumor cells within 4 h after its injection [232].

Coelho et al. investigated the conjugation of bortezomib with pegylated gold NPs (PEGAuNPs), in vitro, on pancreatic and lung cancer cells and reported that this conjugation increased the inhibitory effect of the drug on cancer cells and decreased its toxicity in normal cells [233].

Another chemotherapeutic drug, afatinib, which was approved for the treatment of lung cancer, was conjugated in vitro with AuNPs to improve its efficacy and biocompatibility in cancer cells [233]. This conjugation significantly inhibited the proliferation of lung cancer cells. Additionally, the alveolar epithelial type I cells maintained their viability and released less pro-inflammatory cytokines when compared to the unconjugated drug [234].

Furthermore, Ramalingam et al. investigated the conjugation of doxorubicin on the surface of AuNPs with polyvinylpyrrolidone for the treatment of lung cancer. They reported a targeted release of the chemotherapy drug, with a strong inhibition of lung cancer cell-growth compared to the unconjugated doxorubicin [235].

Several studies also focused on obtaining NPs by the use of natural compounds that might prove beneficial in the treatment of different types of cancer. For instance, Balakrishnan et al. developed AuNPs conjugated with quercetin, and reported their role in inhibiting the migration, invasion, angiogenesis and metastasis of breast cancer cells [236]. Additionally, another study conducted by Chen et al. demonstrated the inhibition of breast cancer MDA-MB-231 cells by AuNPs capped with gallic acid [237].

The analysis of cancer cell morphology indicated that AgNPs can cause cell death. Due to their properties, magnetic AgNPs of various sizes were successfully used to target breast cancer cells (SKBR3) and floating leukemia cells (SP2/O), as produced by Jun et al. [238].

On the other hand, AgNPs coated with folic acid and conjugated with doxorubicin were produced by Wang et al., with cell death occurring after 8 h [239]. In one other study, Fang et al. obtained polyconjugated AgNPs with tertiary amines, doxorubicin, guanidines and imidazoles, that were responsible for increasing their intracellular uptake and cytotoxicity in lymphoma cells [240].

AshaRani et al. investigated the cellular and molecular mechanisms induced by AgNPs using IMR-90 normal human lung tissue and the U251 glioblastoma cell line. It was discovered that the synthesized NPs were able to adsorb on their surface proteins from the cytosol, proteins that can influence the function of intracellular factors and can regulate gene expression and pro-inflammatory cytokines [241]. One study even showed that AgNPs could alter the regulation of more than 1000 genes [242]. Recently, it has also been demonstrated that the autophagy of cancer cells is another mechanism induced by such materials [243].

Nonetheless, AgNPs synthesized from plant extracts showed a more pronounced cytotoxic effect on lung carcinoma cells (A549) than on healthy lung cells, indicating that the AgNPs could target a cell-specific toxicity, which could be indicated by a lower pH level in cancer cells [244]. Moreover, AgNPs obtained using *E. sylvaticum* proved cytotoxic efficacy against osteosarcoma cell line MG-63 [90].

### 5.3. Angiogenesis Inhibition

The process of new blood vessel formation is also a remarkable opportunity for the use of gold nanoparticles in cancer therapy [245]. By targeting NPs, a variety of therapeutic agents, such as antibodies and small compounds, could be employed as therapeutic solutions [246].

Mukherjee et al. found that AuNPs inhibit angiogenesis by inhibiting the protein phosphorylation in a dose-dependent manner, with almost complete inhibition observed at concentrations in the 335–670 nM range. It has been suggested that the inhibition mechanism involves the direct binding of AuNPs to heparin-binding growth factors [247].

A more recent study on AuNPs biosynthesized with the use of apigenin has also emphasized their potential in angiogenesis suppression, as well as in mediating anti-proliferation of cholangiocarcinoma, thus pointing to their dual activity [248]. Moreover, another novel research study that focused on investigating the effects of sorafenib derivatives-capped AuNPs showed in vitro suppression of angiogenesis, as well as of tumor migration and mesenchymal–epithelial transition [249].

When discussing AgNPs, a study performed by Kalishwaralal et al. demonstrated the inhibition of angiogenesis by nanoparticles biologically synthesized using *Bacillus licheniformis* on bovine retinal endothelial cells (BRECs) [250]. A further investigation demonstrated that AgNPs inhibited VEGF-induced cell proliferation, migration, and tube formation in BRECs, that were used as an in vitro model system [251].

Another study focused on observing the effects posed by eco-friendly synthesized AgNPs with the use of an extract obtained from a brown alga, *Dictyota ciliolate*. Besides enhanced cytotoxicity on a lung adenocarcinoma cell line, the NPs also exhibited antiangiogenic effects, considering the inhibition of tertiary blood vessel development [252].

Moreover, AgNPs synthesized from *Achillea biebersteinii* flower extract at a concentration of 200 g/mL showed a 50% reduction in the newly formed vessels on a rat aortic ring model [253]. One other example is that of AgNPs produced using a *Saliva officinalis* extract that exhibited dose-dependent antiangiogenic properties on a chicken embryo [254]. The inhibitory effect of such nanomaterials on the induced angiogenic activity in human breast cancer cells has also been shown [255].

### 5.4. Antimicrobial Properties

Metal NPs appear to be promising antibacterial agents given their high surface-to-volume ratios, as well as their diverse mechanisms for bacterial inhibition and bactericidal action, which decreases bacterial resistance. Silver, copper, gold, titanium, magnesium, and zinc NPs demonstrated the most encouraging results among the metal NPs that were studied [256,257].

Nonetheless, more complex nanoparticles, such as Ag/Cu_2_MoO_4_ NPs, were also shown to possess an inhibitory activity against *Pseudomonas aeruginosa* and *Streptococcus pneumoniae*, as well as the capacity for glucose detection [258], while LaNiO_3_/SrCeO_3_ and NiV_2_O_6_/CeO_2_ nanocomposites showed promising activity against *Klebsiella pneumoniae* and *Bacillus cereus*, and *Streptococcus pneumoniae, Enterococcus faecalis*, *Pseudomonas aeruginosa*, and *Legionella pneumophila*, respectively, as well as photocatalytic activity [259,260]. Moreover, iron- and nickel-introduced bimetallic metal-organic frameworks were also tested on *Mycobacterium tuberculosis* and *Helicobacter pylori*, presenting promising activity [261]. Silver indium sulfide/nickel molybdenum sulfide nanostructures also exhibited antibacterial activity on *Escherichia coli* and *Staphylococcus aureus* under light irradiation, as well as enhanced peroxidase-like activity for the detection of acid uric [262]. Furthermore, NiCo_2_O_4_-Bi_2_O_3_-Ag_2_ZrO_3_ nanocomposites also revealed excellent bactericidal activity against *P. aeruginosa* and *S. pneumoniae*, which proves the multifaceted actions such nanomaterials possess [263].

Metal NPs have an advantage over bulk metals due to their small size and high surface area, which allows for greater interaction with microorganisms. Several mechanisms are responsible for their antimicrobial activity, including oxidative stress, membrane damage, and DNA damage [264].

Oxidative stress is one of the most common mechanisms explaining antimicrobial activity. The ROS produced by these nanoparticles, such as superoxide anions and hydroxyl radicals, can damage the bacterial cell membrane and proteins. For instance, AgNPs have been shown to produce ROS that result in lipid peroxidation of bacterial membranes and inhibition of bacterial growth [265]. Similarly, it has been demonstrated that copper NPs generate ROS that damage the cell membrane and induce oxidative stress, leading to bacterial cell death [266].

Membrane damage is another mechanism of antimicrobial activity. Metal NPs can interact with bacterial membranes and compromise their integrity, resulting in intracellular component leakage and ultimately cell death. It has been demonstrated, for instance, that AuNPs penetrate the bacterial membranes and change their permeability, eventually resulting in cell lysis and bacterial death [267]. It has also been shown that AgNPs, as well as zinc oxide NPs, adhere to and penetrate bacterial membranes, altering their properties [268,269].

Lastly, metal NPs can cause DNA damage, which results in cell death or mutations. These particles can enter bacterial cells and interact with DNA, causing strand breaks and other forms of damage. It has been established, for instance, that AgNPs induce DNA damage, resulting in decreased bacterial viability [270].

Sondi and Salopek-Sondi revealed the excellent antimicrobial action of AgNPs against *Escherichia coli* cultures treated with AgNPs that accumulated in the cell wall and caused the formation of gaps in bacterial walls, thus resulting in cell death [271,272].

Various kinds of AgNPs with an average size of around 15 nm were synthesized, and all of them exhibited promising antimicrobial and bactericidal activity against both Gram-positive and Gram-negative bacteria, including multi-resistant strains such as methicillin-resistant *Staphylococcus aureus* [273,274]. One other study investigated the effectiveness of AgNPs against *E. coli* and *S. aureus* and yeast. The obtained results indicated complete inhibition of *E. coli* and yeast at a low concentration of AgNPs, while only a minor effect was noted on *S. aureus* [275].

Since antibiotic resistance has become a major threat to public health, there is a need for novel and innovative methods to combat bacterial infections. In the field of antimicrobial therapy, the association of NPs with antibiotics has become a significant area of research [276]. Such an association has several advantages over using antibiotics alone.

First of all, it can increase the antimicrobial activity of antibiotics by enhancing their effectiveness against bacterial pathogens. It has been demonstrated that combining AgNPs with antibiotics such as amoxicillin and tetracycline enhances their antibacterial activity against *E. coli*, *S. aureus* and *Salmonella typhimurium* resistant strains [277,278]. Similarly, it has been demonstrated that the association of AuNPs with antibiotics such as streptomycin or azithromycin increases their antibacterial activity against *Pseudomonas aeruginosa* [279,280].

The combination of NPs and antibiotics can also boost the drug’s bioavailability and decrease its toxicity. Antibiotics can be carried by NPs, allowing for a targeted delivery to the site of infection. Consequently, the risk of toxicity and side effects is reduced [281].

Thirdly, this association can also inhibit the emergence of antibiotic resistance. NPs can function as synergistic agents, enhancing the antibacterial activity of antibiotics and decreasing the likelihood of antibiotic resistance. It has been shown that the combination of AgNPs with antibiotics inhibits the development of resistance in *E. coli* and *S. aureus* [282].

Likewise, AgNPs biologically synthesized using *Klebsiella pneumoniae* culture supernatant increased the efficacy of various antibiotics, such as amoxicillin, clindamycin, erythromycin, penicillin G, and vancomycin against *S. aureus* and *E. coli* [283].

Regarding the antifungal properties of nanomaterials, fungus-mediated synthesized AgNPs showed enhanced activity against *Phoma glomerata*, *P. herbarum*, *Fusarium semitectum*, *Trichoderma* sp. and *Candida albicans,* especially when associated with fluconazole [284]. Moreover, an antimicrobial gel formulation containing AgNPs also displayed good antifungal activity against strains of *Aspergillus niger* and *Candida albicans* [285].

One other study showed that AgNPs synthesized using ribose and stabilized by sodium dodecyl sulfate highlighted a good antifungal activity against *Candida albicans* and *C. tropicalis*, comparable to that of conventional antifungals, such as amphotericin B [286].

Other examples of biosynthesized AgNPs with promising antifungal activity, especially on *Candida albicans,* are those obtained using plants such as *Brassica oleracea*, *Camellia sinensis*, *Glycine max*, *Mentha piperita*, and *Tagetes erecta* [103,287,288,289].

Nevertheless, AuNPs can also present antifungal activity. For example, nanoparticles synthesized using the solvothermal method show size-dependent antifungal activity against *Candida* isolates, with smaller sized particles (7 nm) exhibiting greater fungicidal activity compared to larger ones (15 nm) [290]. Moreover, AuNPs biosynthesized using olive leaf extract and *Allium sativum* also presented good antifungal activity on *Candida* species, which was mainly due to ROS formation [291,292].

### 5.5. Antiviral Action

Due to their unique physicochemical properties, such as small size, high surface area, and catalytic activity, metal nanoparticles have emerged as a promising group of antiviral agents. These characteristics allow them to interact with viral components (e.g., proteins and membranes) and to disrupt their structure or function, thereby preventing viral replication and transmissibility [293].

The antiviral activity of AgNPs against a wide variety of viruses, such as herpes simplex virus (HSV), HIV-1, and influenza virus, has been extensively studied [294,295]. By interfering with viral envelope proteins or RNA, AgNPs have been shown to prevent viral entry, replication, and release. Moreover, by inducing production of cytokines and chemokines that activate antiviral defenses, AgNPs can also boost the immune response [296]. Due to the varying methods of synthesis and sizes of these NPs, it is extremely difficult to determine their precise mechanism of action. The research conducted by Gaikwad et al. confirmed that the smaller their size, the greater their inhibition efficacy [297]. Although the antiviral mechanism remains somewhat unclear, it appears that AgNPs interact with viral surface proteins to prevent their penetration or to destroy them, thereby affecting the structure and integrity of the virion. For instance, a study conducted in vitro on AgNPs revealed that they prevented the SARS-CoV-2 virus from entering cells and thereby prevented infection [298].

The anti-HIV activity was demonstrated by Lara et al. at an early stage of viral replication. Polyvinyl pyrrolidone (PVP)-coated AgNPs inhibited both HIV-1 cell-free and cell-associated infection and showed that, as virucidal agents, they prevent the infection regardless of viral tropism or resistance profile, bind to envelope glycoprotein gp120 and therefore prevent CD4-dependent virion binding and infectivity. Therefore, AgNPs proved to be effective inhibitors of HIV, as well as of HBV [299,300,301].

A study involving the intranasal administration of AgNPs to mice revealed that the tested sample had a significant effect on survival, as evidenced by the decrease in lung viral titer levels, minor pathological lesions in the lung tissue, and a remarkable survival advantage after H3N2 influenza virus infection [302]. The size and zeta potential of the biologically synthesized AgNPs were responsible for inhibiting the viability of HSV types 1 and 2 and human parainfluenza virus type 3 [297].

The treatment of Vero cells with non-cytotoxic concentrations of AgNPs also significantly inhibited the Peste des petits ruminants virus replication. The mechanism on viral replication is due to the interaction of AgNPs with the surface and core of the virion [303].

AuNPs have also demonstrated antiviral activity against multiple viruses, such as hepatitis B virus, human papillomavirus, human rhinovirus and even SARS-CoV-2 [304,305]. One recent study also demonstrated that AuNPs inhibited the influenza virus’ pre- and post-entry life-cycle phases, implicitly providing promising antiviral activity [306]. Nevertheless, AuNPs’ mechanism of action is not fully understood, but it is believed to involve disruption of viral attachment and entry into host cells, as well as the inhibition of viral replication and gene expression [307]. Moreover, such particles could also prove to be useful for virus detection applications [308].

Even though metal NPs hold great promise as antiviral agents, there are still obstacles to overcome, such as optimizing their size, shape, and surface for maximum efficacy and minimizing their potential toxicity to host cells. In spite of this, their unique properties and broad-spectrum activity make them a promising option for the development of novel antiviral therapies [309].

The antiviral action of other different types of NPs was reported by Murugan et al. against the Dengue virus, and by Gutierrez et al. against rotavirus [310,311]. Using in vitro methods, Kumar et al. investigated the antiviral action of synthesized FeNPs on the H1N1 influenza A virus, and obtained promising results [312]. Another molecular study concluded that iron oxide NPs effectively interacted with the viral proteins of SARS-CoV-2 and the hepatitis C virus and prevented the penetration and binding in the host cells. This finding was proposed to the FDA for inclusion in clinical trials [313].

### 5.6. Anti-Inflammatory Activity

Inflammation is an immunological response sustained by the production of pro-inflammatory cytokines, the activation of the immune system, and the release of prostaglandins and chemotactic substances, including complement cells, interleukin-1 (IL-1), TNF-α and TGF-β [314,315].

Metal NPs have emerged as a promising material class due to their potential anti-inflammatory properties. According to numerous studies, such nanomaterials can suppress the production of pro-inflammatory cytokines, chemokines, and ROS to reduce inflammation. These characteristics make them promising therapeutic candidates for the treatment of a variety of inflammatory diseases [316,317,318,319].

The ability of AgNPs to inhibit the production of pro-inflammatory cytokines, such as TNF-α and IL-1 in macrophages, has been attributed to their potent anti-inflammatory activity. In addition, AgNPs reduce the expression of the inducible nitric oxide synthase (iNOS) enzyme, which plays a crucial role in the inflammatory response [320].

AuNPs inhibit the production of pro-inflammatory cytokines and chemokines, such as interleukin-6 (IL-6), interleukin-8 (IL-8), and monocyte chemoattractant protein-1 (MCP-1) in human endothelial cells, according to several studies [321,322,323,324].

Moreover, copper NPs have also exhibited anti-inflammatory activity by inhibiting the production of pro-inflammatory cytokines such as IL-1, TNF-α, and IL-6 in lipopolysaccharide (LPS)-stimulated macrophages, whereas zinc oxide nanoparticles (ZnO NPs) have been found to inhibit the activation of nuclear factor-kappa B (NF-kB) [325,326].

The anti-inflammatory activity of AgNPs produced using ginger oil was tested via an inhibition of albumin denaturation assay. The synthesized particles exhibited very good anti-inflammatory activity, with 90% of inhibition at a dose of 60 μL [327]. In another study, AgNPs and AuNPs obtained using *Prunus serrulata* fruit extract demonstrated in vitro anti-inflammatory properties, with a significant suppression of lipopolysaccharide-induced activation of the NF-κB signaling pathway via p38 MAPK on a RAW 264.7 murine macrophage cell line [328].

This anti-inflammatory activity was also highlighted in in vivo experiments. For instance, one study reported anti-inflammatory activity in rats treated intracolonically or orally with nanocrystalline silver (NPI 32101), showing substantially reduced colonic inflammation and rapid dose-dependent healing [329].

AgNPs that were delivered topically on wound mice models, and showed significant antimicrobial properties, also displayed a decrease in wound inflammation. Therefore, using both in vitro and in vivo models, Wong et al. discovered that AgNPs were able to reduce inflammatory markers, indicating that such particles could suppress inflammatory effects in the early stages of wound-healing [330].

AgNPs treatment significantly increased apoptosis in inflammatory cells and decreased the level of proinflammatory cytokines in a porcine model of contact dermatitis. Biosynthesized AgNPs can also inhibit UV-B-induced cytokine production in cells and reduce edema and cytokine levels in different pig tissues [331,332].

### 5.7. AgNPs and AuNPs in Clinical Trials

Due to their unique features, silver and gold NPs have become potential candidates for a variety of biomedical applications. Recent clinical trials have established the viability of these nanomaterials as antibacterial agents, drug delivery vehicles, and imaging agents for the diagnosis and treatment of a variety of disorders [333,334]. For example, AgNPs have demonstrated significant promise in wound-healing, whereas AuNPs have been exploited in cancer diagnosis and treatment (Table 3) [335,336].

However, other types of metal particles, such as iron oxide NPs, have attracted significant attention in recent years due to their unique magnetic properties, biocompatibility, and versatile applications in clinical settings. Their properties have been extensively studied in preclinical and clinical trials for various biomedical applications, including magnetic resonance imaging (MRI), drug delivery, and hyperthermia therapy [337].

Overall, metal NPs hold enormous potential for the creation of new and successful treatments, but their clinical applications must be examined and monitored with considerable care.

## 6. Cytotoxicity of Metal NPs

The adverse effects of nanomaterials on human organs have gradually been noticed, and may have an impact on their use in biomedical applications [338,339]. A metal-based NP can easily cross cell membranes due to its size comparable to that of protein molecules. As a result, they can interact with proteins or subcellular organelle structures, leading to neurotoxicity, immunotoxicity, and genotoxicity. Implicitly, the biosafety of such materials and technologies has received significant attention considering their widespread use [339].

AgNPs can be ingested, injected intravenously, or absorbed through the skin. The absorbed AgNPs are distributed in numerous organs and systems, including the respiratory tract, digestive and urinary systems, and nervous system, as well as the immune and reproductive systems, but primarily in the spleen, liver, kidney, and lungs, with a lesser deposition in the teeth and bones [338,340]. The non-specific distribution of AgNPs can result in skin, eye, respiratory, and reproductive system toxicity and even neurotoxicity, thereby restricting their application.

The potential cytotoxicity of AgNPs depends on properties such as size, shape, and concentration, and routes of administration [341]. For instance, Wang et al. employed TEM and the integration of synchrotron radiation beam transmission X-ray microscopy (SR-TXM) with 3D tomographic imaging to collect data on the uptake, buildup, cellular degradation, chemical transformation, and removal of AgNPs at a cellular level. The experiment demonstrated that the cytotoxicity was caused by the chemical transformation of AgNPs to Ag+, Ag-O- and Ag-S- species, which could induce biological modifications [342].

Similarly, the cytotoxicity of AuNPs has been examined and reviewed by several researchers [343,344,345]. Different routes of administration can have varying effects on the biodistribution of drug carriers [346]. The in vivo distribution after administration largely depends on the NPs’ size, surface charge and surface hydrophobicity. The influence of these factors on NPs uptake by the mononuclear phagocyte system has also been described [347].

The size-dependent permeability of AuNPs through the skin and intestine has been demonstrated [348]. AuNPs of different sizes (15, 50, 100, and 200 nm) were administered intravenously to mice, and their biodistribution was studied. AuNPs of all sizes primarily accumulated in the liver, lungs, and spleen, whereas the accumulation in other tissues was dependent on the size of AuNPs. Large amounts of 15 nm AuNPs were detected in all tissues, including blood, liver, lungs, spleen, and kidney, and it was discovered that they could cross the blood-brain barrier, similar to the 50 nm AuNPs. On the other hand, only a small amount of the 200 nm AuNPs could be found in blood, brain, stomach, and pancreas [349].

Moreover, the shape also poses an important influence of the observed toxicity. For instance, AuNPs stars were the most cytotoxic type of investigated particle, whereas AuNPs spheres seem to be safest, but had a reduced anticancer potential [350].

At the same time, iron oxide NPs are associated with a low toxicity in the human body [351,352,353,354]. For example, an in vitro study comparing several metal oxide nanoparticles showed that iron oxide ones are not cytotoxic at any concentration smaller than 100 mg/mL [355]. Nevertheless, the absence of cytotoxicity does not guarantee that they do not pose a risk to human health in specific applications, as several recent studies have reported potentially negative cellular effects, such as DNA damage, and mitochondrial membrane dysfunction, as well as changes in gene expression [356,357].

Among the several processes that might be linked to the toxicity of NPs in the body, it appears that magnetic iron oxide NPs’ toxicity is caused by excessive ROS production [358,359,360,361]. High levels of ROS can damage the cells through lipid peroxidation, mitochondrial damage, DNA disruption, modulation of gene transcription and protein oxidation, which can trigger mechanisms that result in decreased physiological functions and cell apoptosis [362]. Other toxic effects include cell-cycle changes and the induction of apoptosis, and can occur in various cell types after iron oxide NPs treatment [363,364].

The cytotoxicity of NPs on normal cells has significant implications for their use in various applications, such as medicine and consumer goods. The aspects described in this section emphasize the need for careful consideration of the potential health risks of NPs and the creation of safer alternatives. For example, since surface coating is one of the most popular modification strategies to reduce the potential toxicity of metal NPs, coating AgNPs with biocompatible materials, such as PEG, could help reduce their cytotoxicity on normal cells [365].

Furthermore, the non-biodegradability of metal NPs raises significant environmental concerns due to their persistence and potential long-term impact on ecosystems. To address this issue, researchers are actively exploring various strategies. One approach involves the development of biodegradable coatings or encapsulation techniques, thus enabling controlled release and gradual degradation over time [366].

Consequently, complex further studies in the area of metal NPs are highly recommended. The first step in ensuring safe clinical integration is a thorough investigation of biocompatibility and toxicity profiles. A second way to improve stability and targeting-accuracy is by developing synthesis methods and surface modifications. Moreover, a deeper comprehension of interactions between biological systems at a molecular level is required [367].

## 7. Key Challenges

Metal NPs represent a fascinating class of nanomaterials with diverse and promising applications across numerous fields. However, as previously mentioned, their unique properties also bring forth several key challenges that must be overcome to fully exploit their potential. One of the foremost challenges is the precise control of their size and shape during synthesis. Achieving uniformity in size and shape is critical for tailoring their properties and functionalities. This often demands complex synthesis techniques and a deep understanding of the underlying chemistry and physics involved. Moreover, scaling up the production of well-defined metal NPs while maintaining this control can be challenging and costly [368].

Another critical issue is the tendency of metal NPs to agglomerate or aggregate, leading to reduced stability and diminished performance. The forces driving agglomeration, such as van der Waals forces and electrostatic interactions, must be mitigated through strategies like surface functionalization or the use of stabilizing agents [369].

In biomedical applications, the toxicity of certain metal NPs, particularly heavy metals like cadmium or mercury, poses a significant challenge. Understanding the potential health risks and developing safe handling procedures is essential for their use in drug delivery, imaging, and diagnostics.

Furthermore, the long-term stability and behavior of such materials under different environmental conditions remain complex and not fully elucidated. Variations in temperature, pH, and exposure to other chemicals can alter their properties, potentially rendering them less effective or even hazardous. Consequently, robust characterization and predictive models are needed to ensure consistent performance.

Lastly, the scalability of production and the environmental impact of their waste disposal are pressing concerns. Large-scale production methods need to be developed that are cost-effective and environmentally sustainable. Additionally, strategies for recycling or disposing of used NPs in an eco-friendly manner are crucial to minimize their ecological footprint [370].

Addressing these challenges demands an interdisciplinary collaboration among material scientists, biologists, clinicians, engineers, and regulatory bodies. As research continues to advance, the potential benefits of metal nanoparticles in catalysis, electronics, and various medical applications make overcoming these hurdles all the more imperative. Ultimately, solving these challenges will not only unlock the full potential of metal NPs, but also ensure their safe and responsible utilization in a wide range of innovative technologies [367].

## 8. Conclusions

Within the last two decades, nanotechnology has continuously and rapidly evolved, since the emergence of multiple methods of obtaining and using raw materials for nanostructures in a variety of fields such as medicine, pharmacy, agriculture, electronics, and many others. 

Due to their unique physicochemical and biological features, silver and gold NPs have been the focus of substantial study in the field of nanomedicine. Their exceptional biocompatibility, stability, and comparatively low toxicity make them attractive candidates for a variety of biological applications. In addition, AgNPs have demonstrated outstanding antibacterial activity against a broad spectrum of pathogens, making them particularly interesting for application in medical device coatings, wound-healing, and water-purification. In contrast, AuNPs have remarkable optical and electrical capabilities, rendering them valuable for imaging, sensing, and drug delivery applications. Several clinical trials have also included such representatives to better understand their implications in various conditions.

Nevertheless, the toxicity, long-term stability, and non-biodegradability of metal NPs represent key issues to weigh when considering their use. Resolving these challenges necessitates interdisciplinary cooperation and robust regulatory oversight to harness the full potential of metal NPs in biomedical applications, while ensuring safety and sustainability. Overall, this review emphasizes the importance and prospects of metal NPs in the biomedical field, considering some of the most recent studies. However, further research is encouraged in order to fully understand their safety and efficacy in clinical settings and to optimize their physicochemical properties for specific applications.

## Figures and Tables

**Table 2:** Advantages and limitations of using biological agents for NPs synthesis.

Biological Material	Advantages	Ref.	Limitations	Ref.
**Plants**	natural source of antioxidant compounds	[95]	the amount of material depends on seasonality of plants the quality of NPs depends on the growth, harvesting conditions and geographical distribution of plants	[130]
	easy to grow and safe to handle	[100]		
	biocompatibility	[96]		
	wide range of applications in different industries and medicine	[98]	specific process conditions such as temperature, pressure, reaction time, chemical reagents additions variation in NPs quality due to irregular shape and size	[59]
	small particle size and zeta potential indicate good stability enhanced biodistribution, efficacy and safety for NPs compared to raw extracts	[130]		
	biodegradable post-materials non-toxic products, safer solvents and reaction conditions	[97]		
	waste prevention	[100]	possible neurotoxicity, tissue toxicity only a few are approved by EMA and FDA, thus needing further analyses.	[131]
	good antifungal activity and promising antibacterial activity on both Gram-positive and Gram-negative strains	[99]		
	promising results in cancer therapy.	[100]		
**Microorganisms**	very good yield due to faster processes environmental safety	[132]	tedious purification steps poor/lack of understanding of the competitive antimicrobial mechanisms challenges in translating the production of NPs to industrial scale potentially diminished yield caused by the growth rate of bacteria, enzymatic activity, biochemical pathways and purification processes.	[133]
	cost effectiveness the preferred material consists of bacteria/fungi due to their capacity for producing higher concentrations of reductase enzyme	[134]		
	ease of cultivation and adjustment of size, shape and morphology of NPs effective penetration through matrix biofilm produced by pathogenic agents, NPs play the role of a “Trojan horse” for bacterial cells.	[134]		
**Algae**	renewable and sustainable natural resources natural products and biodegradable post-materials	[135]	limited applicability, low biocompatibility and, in some cases, elevated toxicity no clear reaction mechanism during NPs formation	[136]
	biocompatibility cost-effectiveness versatility	[137]		
	scalability reduced negative environmental impact.	[138]	irregular shapes and sizes depending on solvents, vehicle, pH, exposure time and age of algal extract.	[59]

**Table 3:** Lists of clinical trials of silver and gold NPs.

NPs Type	Study Title	Material	Targeted Condition	Clinical trials.gov Identifier
**AgNPs**	Topical Silver Nanoparticles for Microbial Activity	Topical cream containing silver NPs	Fungal foot infection Bacterial infection	NCT03752424
	Topical Application of Silver Nanoparticles and Oral Pathogens in Ill Patients	Innocuous gel compound with 12 ppm of AgNPs	Dental diseases	NCT02761525
	Silver Nanoparticles in Multidrug Resistant Bacteria	AgNPs	Multidrug resistant bacteria	NCT04431440
	Nanosilver Fluoride to Prevent Dental Biofilms Growth	Nanosilver fluoride	Dental caries	NCT01950546
	Remineralization of Dentine Caries Using Two Remineralizing Agents Which Are Nanosilver Fluoride and Casein phosphopeptides amorphous Calcium Phosphate	Nanosilver fluoride vs. sodium fluoride with casein phosphopeptides amorphous calcium phosphate on carious lesion	Dental caries	NCT04930458
	Radiographic Assessment of Glass Ionomer Restorations with and Without Prior Application of Nano Silver Fluoride in Occlusal Carious Molars Treated with Partial Caries Removal Technique	Nano silver fluoride solution	Partial dentin caries removal	NCT03193606
	Clinical Evaluation of Silver Nanoparticles in Comparison to Silver Diamine Fluoride in Management of Deep Carious Lesions	AgNPs vs. silver diamine fluoride	Caries, dental therapy	NCT05231330
	Addition of Silver Nanoparticles to an Orthodontic Primer in Preventing Enamel Demineralization Adjacent Brackets	AgNPs incorporated into the primer orthodontic Transbond XT	Tooth demineralization	NCT02400957
	Evaluation of Antimicrobial Efficacy and Adaptability of Bioceramic Sealer Containing Nanoparticles	Bioceramic sealers with classic mix vs. AgNPs vs. chitosan	Endodontic disease	NCT04481945
	Silver Nanoparticle Investigation for Treating Chronic Sinusitis	Topical colloidal silver	Chronic rhinosinusitis	NCT03243201
	The Antibacterial Effect of Nanosilver Fluoride on Primary Teeth	Topic solution of silver fluoride NPs	Microbial colonization for preventing dental caries in children	NCT05221749
	Colloidal Silver, Treatment of COVID-19	Inhalation colloidal silver solution	SARS (severe acute respiratory syndrome)	NCT04978025
	Evaluation of Silver Nanoparticles for the Prevention of COVID-19	AgNPs solution with 1 wt% concentration (0.6 mg/mL metallic silver) for mouthwash and nose rinse	Coronavirus disease (COVID-19)	NCT04894409
	SARS-CoV-2-Spike-Ferritin-Nanoparticle (SpFN) Vaccine with ALFQ Adjuvant for Prevention of COVID-19 in Healthy Adults	SpFN COVID-19 vaccine with Army Liposomal Formulation QS21 (ALFQ) adjuvant	SARS-CoV-2 infection	NCT04784767
	Fluor Varnish with Silver Nanoparticles for Dental Remineralization in Patients with Trisomy 21	Fluor dental varnish plus 25% 50 nm AgNPs vs. fluor dental varnish	Dental remineralization, Down syndrome	NCT01975545
	Comparison of Central Venous Catheters with Silver Nanoparticles Versus Conventional Catheters	Central venous catheter impregnated with AgNPs (AgTive^®^)	Central venous catheter related infections	NCT00337714
	Evaluation of Diabetic Foot Wound Healing Using Hydrogel/Nano Silver-based Dressing vs. Traditional Dressing	Hydrogel/nano silver-based dressing	Diabetic foot wound	NCT04834245
	Efficacy of Silver Nanoparticle Gel Versus a Common Antibacterial Hand Gel	Nano-silver gel exposed vs. alcohol-based hand gel	Antibacterial activity in normal patients’ skin	NCT00659204
	Post-operative Pain Reduction	Conventional calcium hydroxide paste vs. combined calcium hydroxide with AgNPs	Effects of the elements	NCT04338633
	A Phase I/II Double-blind Safety and Efficacy Evaluation of Nowarta110 in Patients with Plantar Warts	Nowarta 110, a colloidal silver fig extract for topical administration	Plantar warts	NCT02338336
	Research on the Key Technology of Burn Wound Treatment	Nano-silver ion gel and dressings for wounds	Burn wounds	NCT03279549
	Altrazeal Range of Motion Study Comparing with Typical Carboxymethyl	Hydrogel nanoparticle wound dressing	Burn wounds	NCT01062191
	Assessment of Postoperative Pain After Using Various Intracanal Medication in Patients with Necrotic Pulp	AgNPs in gel form vs. calcium hydroxide intracanal medication	Postoperative pain	NCT03692286
	Preadmission Skin Wipe Use for Surgical Site Infection Prophylaxis in Adult Orthopaedic Surgery Patients	Bath wipes impregnated with multiple ingredients, among which colloid silver	Surgical site infection	NCT03401749
	Thyme and Carvacroll Nanoparticle Effect on Fungi	Thyme and carvacrol AgNPs	Aspergillosis	NCT04431804
	Clinical and Radiographic Evaluation of the Synergistic Effect of Nano Silver Particles and Calcium Hydroxide Versus Triple Antibiotic Paste as Antibacterial Agents for Lesion Sterilization and Tissue Repair (LSTR) in Necrotic Primary Molars	Nano-silver particles and calcium hydroxide vs. triple antibiotic paste	Root canal infection	NCT05681221
	Effect of Metallic Nanoparticles on Nosocomial Bacteria	AgNPs and CuNPs	Nosocomial infections	NCT04775238
	Evaluation of the Antimicrobial Fiber Reinforced Composite Resin Space Maintainer Modified With Silver Nano Particles	Fiber reinforced composite resin space maintainer modified with silver nano particles	Dental caries in children	NCT05902975
**AuNPs**	Gold Factor on Knee Joint Health and Function	Dietary supplement: Gold factor (AuNPs)	Knee arthritis Knee osteoarthritis Knee pain chronic Knee discomfort Rheumatoid arthritis Knee pain swelling	NCT05347602
	Diagnostic and Prognostic Accuracy of Gold Nanoparticles in Salivary Gland Tumors	CD24-Gold nanocomposites	Carcinoma ex pleomorphic adenoma of salivary glands Pleomorphic adenoma of salivary glands	NCT04907422
	Plasmonic Photothermal and Stem Cell Therapy of Atherosclerosis Versus Stenting	Stenting and micro-infusion of NP	Coronary artery disease Atherosclerosis	NCT01436123
	Enhanced Epidermal Antigen Specific Immunotherapy Trial -1	C19-A3 GNP drug	Type 1 diabetes	NCT02837094
	NU-0129 in Treating Patients with Recurrent Glioblastoma or Gliosarcoma Undergoing Surgery	NU-0129 drug	Gliosarcoma Recurrent glioblastoma	NCT03020017
	Plasmonic Nanophotothermal Therapy of Atherosclerosis	Transplantation of NPs	Stable angina Heart failure Atherosclerosis Multivessel coronary artery disease	NCT01270139
	Effect of Nano Care Gold on Marginal Integrity of Resin Composite	Nano Care Gold (silver and gold NPs) suspended in 70% isopropyl alcohol, for cavity pre-treatment.	Caries class ii	NCT03669224
	A Phase-I Study of a Nanoparticle-based Peptide Vaccine Against Dengue Virus	Nanoparticle-based peptide vaccine	Dengue fever	NCT04935801
	A Phase-I Study of a Nanoparticle-based Peptide Vaccine Against SARS-CoV-2	Nanoparticle-based peptide vaccine	Coronavirus SARS-CoV-2 infection COVID-19	NCT05113862
	Diagnosis of Gastric Lesions from Exhaled Breath and Saliva	Chemical nanosensors based on organically functionalized AuNPs and carbon nanotubes	Stomach diseases	NCT01420588
	Exploratory Study Using Nanotechnology to Detect Biomarkers of Parkinson’s Disease from Exhaled Breath	Combinations of nanomaterial-based sensors	Parkinson’s disease Parkinsonism	NCT01246336

## Data Availability

Not applicable.
